# Deep Vein Thrombosis after Femoral Arterial Access: Pathophysiologic and Therapeutic Challenges

**DOI:** 10.1155/2019/1849256

**Published:** 2019-09-08

**Authors:** Evan Harmon, Yoo Jin Lee, Sula Mazimba, Kanwar Singh, Aditya Sharma, Younghoon Kwon

**Affiliations:** University of Virginia Health System, Charlottesville, VA, USA

## Abstract

Deep vein thrombosis (DVT) after femoral arterial access is a rare complication of left heart catheterization (LHC). The reasons for paradoxical venous clot formation after arterial access are identifiable in some cases but less clear in others. Here, we present one case of provoked DVT after femoral access followed by a second case in which clot formation appears to be spontaneous. Additionally, though each of the patients presented here demonstrated thrombus resolution, only one received anticoagulation. These cases highlight the complex pathophysiology of DVT following femoral arterial access and the challenges of management strategy selection.

## 1. Introduction

Femoral access site complications following left heart catheterization (LHC) or coronary angiography are well described and include arterial thrombosis, hematoma (both femoral and retroperitoneal), pseudoaneurysm, and arteriovenous fistula. Another vascular femoral access site complication that is less often encountered is deep vein thrombosis (DVT). The few reports of DVT after femoral arterial access in the published literature have also cited one or more other provoking factors which could explain venous thrombus formation. However, a subset of patients without overt DVT risk factors appear to suffer DVT after femoral arterial access for reasons that remain unclear.

## 2. Case Descriptions

### 2.1. Case 1

An 83-year-old man with a history of rheumatic mitral stenosis (MS) and atrial fibrillation underwent preoperative cardiac catheterization with the artery and venous access obtained under direct ultrasound visualization without difficulty using a 6 Fr sheath and 7 Fr sheath, respectively, for concurrent left and right heart catheterization. Femoral access was chosen given recent computed tomography (CT) angiogram of the chest, abdomen, and pelvis demonstrating extremely tortuous supra-aortic vessels, complicating radial access. Upon completion of his catheterization procedure, the arterial sheath was removed once ACT was below 180 seconds. Per standard catheterization laboratory protocol, firm manual compression with full pressure was applied for five minutes, followed by gentler pressure for five minutes until hemostasis was visually ascertained. An intermittent pedal pulse check confirmed distal perfusion during this time. In the immediate postcatheterization period, the patient was noted to have a small right groin hematoma which resolved with an additional few minutes of direct compression.

**Figure 1:**
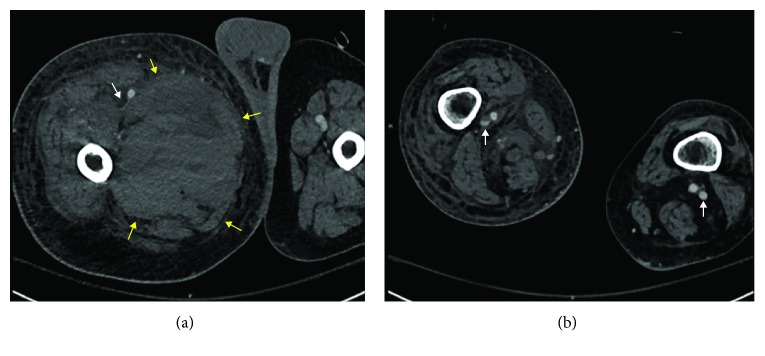
(a) Collapsed right-sided common femoral vein (white arrow) due to massive hematoma (yellow arrows). (b) Nonocclusive thrombus in right-sided midpopliteal vein, compared to patient left-sided midpopliteal vein (white arrows).

Five days later, the patient presented to the emergency department with progressive right thigh pain, ecchymoses, swelling, and difficulty ambulating. CT scan demonstrated a 14 cm right-sided femoral hematoma, and follow-up CT angiogram revealed a 2.3 × 2.0 × 3.5 cm right profunda femoris proximal branch pseudoaneurysm associated with the hematoma. This study also demonstrated severe stenosis of the right superficial femoral vein secondary to extrinsic compression with nonocclusive wall adherent thrombus in the right midpopliteal vein (Figure 1).

Coil embolization of his pseudoaneurysm was attempted, but two hours post procedure, the patient demonstrated severe motor and sensory deficits in the right lower extremity with associated hemoglobin drop from 8.1 g/dL to 6.0 g/dL. He was taken for emergent surgical evacuation of his right thigh hematoma.

Though his course was further complicated by additional mild, transfusion-responsive bleeding events, his bleeding ultimately stabilized and the patient was successfully transitioned back to his home warfarin dose by the day of discharge to be continued indefinitely in the setting of his known atrial fibrillation. A follow-up ultrasound obtained two weeks after initial identification of the patient's midpopliteal DVT demonstrated thrombus resolution.

### 2.2. Case 2

A 40-year-old man with a history of hypertension and hyperlipidemia was referred for a one-month history of exertional chest pain consistent with typical angina. Cardiac CT demonstrated multivessel disease, and LHC was pursued.

The right femoral artery was accessed using a 6 Fr sheath under direct ultrasound visualization. Following the procedure, an Angio-Seal™ closure device (St. Jude Medical, Minnetonka, Minnesota) was used to achieve hemostasis. Manual compression was still applied for about three minutes. Access site examination was unremarkable, without bruit auscultation or evidence of hematoma.

**Figure 2:**
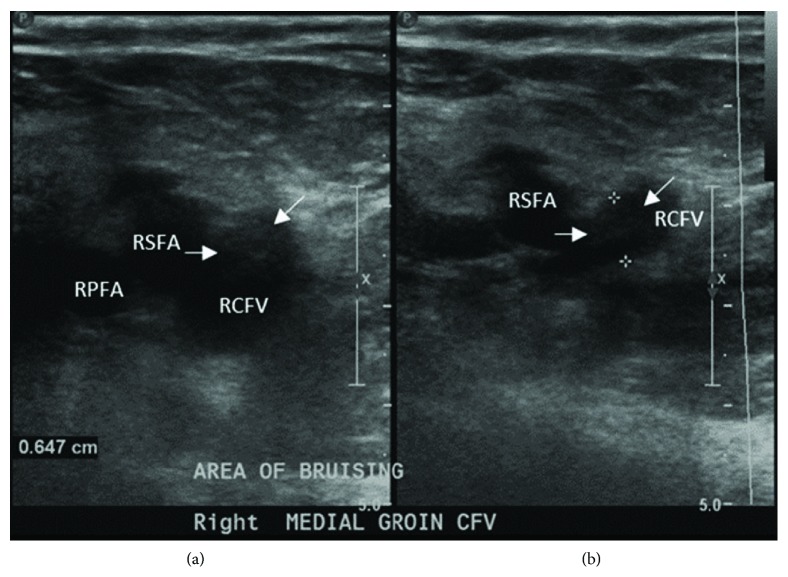
Partially thrombosed (white arrows) right common femoral vein. (a) is without compression; (b) is with compression, which demonstrates incomplete collapse of the RCFV.

**Figure 3:**
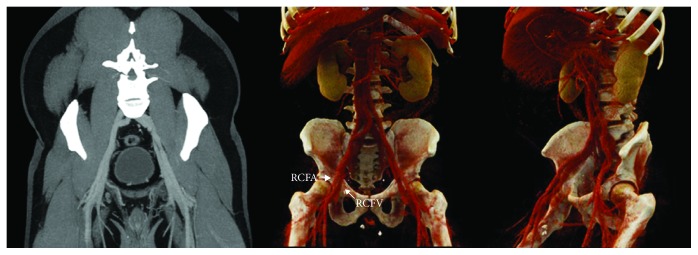
MIP (maximal intensity projection) and 3D cinematic rendering images of the CT venogram performed 1 month after the ultrasound exam were normal, with no evidence of residual venous thrombus or traumatic pseudoaneurysm.

One week after PCI, the patient reported new, worsening groin pain radiating to the abdomen. Ultrasound was ordered for suspected pseudoaneurysm but instead demonstrated nonocclusive thrombus within the right common femoral vein without evidence of hematoma or arterial pathology (Figure 2). Hypercoagulability testing was unrevealing: antithrombin-III function, Protein C activity, Protein S activity, anti-cardiolipin IgM/IgG, and anti-beta-2-glycoprotein-1 IgM/IgG were all within normal limits. Factor V Leiden and prothrombin assays were unremarkable. Serum homocysteine was only mildly elevated at 14 *μ*mol/L (reference range 5-12 *μ*mol/L). Given these findings, three months of oral anticoagulation in addition to the patient's dual antiplatelet regimen was planned, but it was later found that the patient had not taken his oral anticoagulation as instructed. Interestingly, he did report resolution of his groin pain and ipsilateral edema, and follow-up CT venogram one month later revealed thrombus resolution (Figure 3).

## 3. Discussion

DVT following femoral arterial access is considered to be a relatively rare vascular complication of LHC with an estimated incidence of 0.05% [1]. Though radial rather than femoral access is becoming more common for routine LHC, the latter remains critical for procedures requiring larger vascular sheath sizes (and longer compression times) such as intra-aortic balloon pump or percutaneous left ventricular assist device placement. It is also the preferable access modality for patients with coronary artery bypass grafts and hemodialysis arteriovenous fistula [2, 3]. DVT incidence following LHC may therefore be underestimated, though prospective investigations with systematic screening are necessary to objectively confirm.

The majority of published DVT cases following femoral arterial access demonstrate a clear provocation in the form of mechanical deep venous compression by a known hematoma, or by concurrent arterial and venous access, such as in Case 1 [4]. Additionally, the age and relative immobility of the patient described in Case 1 represent at least two additional nonmechanical risk factors for DVT formation. In contrast, Case 2 represents only the second reported case of seemingly unprovoked DVT formation following LHC utilizing femoral arterial access [5]. In such “unprovoked” cases, we suspect a likely inadvertent venous compression during femoral arterial hemostasis as the mechanism for DVT formation. Supporting this hypothesis is the evidence that DVTs in this setting arise almost universally in the proximal rather than distal lower extremity venous system, which is reflected in our two cases. Literature review demonstrates only one other case of distal DVT following LHC [5], suggesting a strong anatomical relationship between the sites of access, hemostasis, and subsequent thrombus formation.

An important question to consider is whether and to what degree the use of a vascular closure device (VCD) in Case 2 may have contributed to DVT formation. To date, large meta-analyses comparing outcomes between manual compression and VCDs have yielded conflicting results. However, the ISAR-CLOSURE trial, which randomized over four thousand patients undergoing coronary angiography to the hemostasis strategies of manual compression, intravascular closure device, or extravascular closure device in a 1 : 1 : 1 fashion, demonstrated that VCDs overall have similar rates of vascular complications compared to manual compression while achieving hemostasis quicker [6]. Among the VCD group, intravascular closure devices such as Angio-Seal™ trended toward significantly fewer access site complications while achieving hemostasis significantly quicker than extravascular devices. These findings would suggest that the use of the Angio-Seal™ closure device in Case 2 is unlikely to explain DVT formation, though an important limitation of the ISAR-CLOSURE trial and others is the lack of inclusion of DVT formation as part of the primary endpoint. One prospective study of over six hundred patients found no difference in minor complications (including DVT) between Angio-Seal™, StarClose™ (extravascular closure device), and manual compression, but additional studies with specific focus on DVT formation following femoral arterial access are needed [7].

Additionally, hypercoagulable workup may be reasonable in select patients in whom the factors contributing to DVT formation following femoral arterial access are not immediately clear. For example, our patient in Case 2 was found to have mild hyperhomocysteinemia, a well-described risk factor for inherent endothelial dysfunction [8]. This finding supports the notion that it is likely the combination of inadvertent arterial compression during hemostasis *and* underlying patient susceptibility which results in seemingly “unprovoked” DVT formation in a subset of cases.

Finally, DVT formation following a percutaneous coronary intervention (PCI) may be therapeutically challenging. In these cases, “triple therapy,” i.e., the addition of oral anticoagulation to dual antiplatelet therapy (DAPT), in the post-PCI setting markedly increases bleeding risk [9]. Nonetheless, current European Society of Cardiology (ESC) guidelines recommend that among most patients with a baseline anticoagulation indication (e.g., atrial fibrillation and mechanical heart valve) undergoing PCI, triple therapy should be continued through at least one month postprocedurally [10]. The most recent CHEST guidelines recommend OAC for three-month duration in the setting of an acute provoked proximal DVT [11]. This regimen was recommended to the patient in Case 2, though he ultimately demonstrated spontaneous resolution of his DVT without anticoagulation. This raises the interesting question of whether less aggressive antithrombotic regimens may be reasonable in select patients found to have DVT following femoral arterial access. Overall, however, these challenges in management should highlight the importance of emphasizing DVT prophylaxis strategies in standard post-LHC management protocols.

In summary, DVT formation is an important potential complication of femoral access LHC. A subset of patients suffer seemingly unprovoked DVT, the pathophysiology of which is unclear but may be attributable to inadvertent venous compression during hemostasis in combination with patient-specific susceptibility factors. Additional research is necessary to guide therapeutic decision-making, particularly in patients requiring DAPT.

## 4. Conclusion

DVT formation is a rare but increasingly reported complication of femoral arterial access LHC. Clot formation in some cases is easily attributable to mechanical venous compression due to arterial complications such as hematoma formation. However, there appears to be a subset of patients in whom DVT formation is unprovoked. The pathophysiology of unprovoked DVT in this setting is unclear but may be attributable to inadvertent venous compression during arterial hemostasis in combination with patient-specific susceptibility factors. Additional research is necessary to not only clearly delineate the mechanism responsible for unprovoked DVT after femoral arterial access but also guide therapeutic decision-making, particularly in patients on DAPT.

## References

[1] Filis K., Arhontovasilis F., Theodorou D. (2007). Management of early and late detected vascular complications following femoral arterial puncture for cardiac catheterization. *Hellenic Journal of Cardiology*.

[2] Michael T. T., Alomar M., Papayannis A. (2013). A randomized comparison of the transradial and transfemoral approaches for coronary artery bypass graft angiography and intervention: the RADIAL-CABG trial (RADIAL versus femoral access for coronary artery bypass graft angiography and intervention). *JACC: Cardiovascular Interventions*.

[3] National Kidney Foundation (2006). Clinical Practice Guidelines for Vascular Access. *American Journal of Kidney Diseases*.

[4] Kim M., Lee J. Y., Lee C. W. (2013). Deep vein thrombosis due to hematoma as a rare complication after femoral arterial catheterization. *Yeungnam University Journal of Medicine*.

[5] Min S. Y., Shin J. H., Lee S. W. (2013). A case of deep vein thrombosis after coronary angiography in a patient using antidepressants and anxiolytics. *Korean Circulation Journal*.

[6] Schulz-Schüpke S., Helde S., Gewalt S. (2014). Comparison of vascular closure devices vs manual compression after femoral artery puncture: the ISAR-CLOSURE randomized clinical trial. *JAMA*.

[7] Yeni H., Axel M., Ornek A., Butz T., Maagh P., Plehn G. (2016). Clinical and subclinical femoral vascular complications after deployment of two different vascular closure devices or manual compression in the setting of coronary intervention. *International Journal of Medical Sciences*.

[8] Eldibany M. M., Caprini J. A. (2007). Hyperhomocysteinemia and thrombosis: an overview. *Archives of Pathology & Laboratory Medicine*.

[9] Staudacher D. L., Kaiser M., Hehrlein C., Bode C., Ahrens I. (2015). Triple antithrombotic therapy after percutaneous coronary intervention (PCI) in patients with indication for oral anticoagulation: data from a single center registry. *PLoS One*.

[10] Valgimigli M., Bueno H., Byrne R. A. (2018). 2017 ESC focused update on dual antiplatelet therapy in coronary artery disease developed in collaboration with EACTS: the Task Force for dual antiplatelet therapy in coronary artery disease of the European Society of Cardiology (ESC) and of the European Association for Cardio-Thoracic Surgery (EACTS). *European Heart Journal*.

[11] Kearon C., Akl E. A., Ornelas J. (2016). Antithrombotic therapy for VTE disease: CHEST guideline and expert panel report. *Chest*.

